# Human iPS cell-derived mural cells as an in vitro model of hereditary cerebral small vessel disease

**DOI:** 10.1186/s13041-020-00573-w

**Published:** 2020-03-19

**Authors:** Yumi Yamamoto, Katsutoshi Kojima, Daisuke Taura, Masakatsu Sone, Kazuo Washida, Naohiro Egawa, Takayuki Kondo, Eiko N. Minakawa, Kayoko Tsukita, Takako Enami, Hidekazu Tomimoto, Toshiki Mizuno, Raj N. Kalaria, Nobuya Inagaki, Ryosuke Takahashi, Mariko Harada-Shiba, Masafumi Ihara, Haruhisa Inoue

**Affiliations:** 1 Research Fellow of Japan Society for the Promotion of Science, Tokyo, Japan; 2 Department of Molecular Innovation in Lipidemiology, National Cerebral and Cardiovascular Center Research Institute, 6-1 Kishibeshinmachi, Suita-shi, Osaka 564-0018 Japan; 3 Department of Diabetes, Endocrinology and Nutrition, Graduate School of Medicine, Kyoto University, 53 Kawahara-cho, Shogoin, Sakyo-ku, Kyoto, 606-8507 Japan; 4 Department of Stroke and Cerebrovascular Diseases, National Cerebral and Cardiovascular Center, 6-1 Kishibeshinmachi, Suita-shi, Osaka 564-0018 Japan; 5 Center for iPS Cell Research and Application (CiRA), Kyoto University, 53 Kawahara-cho, Shogoin, Sakyo-ku, Kyoto, 606-8507 Japan; 6 iPSC-based Drug Discovery and Development Team, RIKEN BioResource Research Center (BRC), Kyoto, Japan; 7 Department of Neurology, Kyoto University Graduate School of Medicine, 53 Kawahara-cho, Shogoin, Sakyo-ku, Kyoto, 606-8507 Japan; 8 Medical-risk Avoidance based on iPS Cells Team, RIKEN Center for Advanced Intelligence Project (AIP), Kyoto, Japan; 9 Department of Degenerative Neurological Diseases, National Institute of Neuroscience, National Center of Neurology and Psychiatry, 4-1-1 Ogawa-Higashi, Kodaira, Tokyo 187-8502 Japan; 10 Department of Dementia Prevention and Therapeutics, Graduate School of Medicine, Mie University, 2-174 Edobashi Tsu, Mie, 514-8507 Japan; 11 Department of Neurology, Kyoto Prefectural University of Medicine, 465 Kajii-cho, Kawaramachi-Hirokoji, Kamigyo-ku, Kyoto, 602-8566 Japan; 12 Neurovascular Research Group, Institute of Neuroscience, Newcastle University, Campus for Ageing & Vitality, Newcastle upon Tyne, NE4 5PL UK

**Keywords:** CADASIL, Notch3, Mural cell, PDGFRβ, Induced pluripotent stem cell, Differentiation, Cerebral small vessel disease

## Abstract

Cerebral autosomal dominant arteriopathy with subcortical infarcts and leukoencephalopathy (CADASIL) is one of the most common forms of hereditary cerebral small vessel diseases and is caused by mutations in *NOTCH3*. Our group has previously reported incorporation of NOTCH3 extracellular domain (N3ECD) in the CADASIL-specific granular osmiophilic materials and increase of PDGFRβ immunoreactivity in CADASIL postmortem brains. Here, we aimed to establish an in vitro model of CADASIL, which can recapitulate those CADASIL phenotypes, using induced pluripotent stem cells (iPSCs). We have refined a differentiation protocol of endothelial cells to obtain mature mural cells (MCs) with their characteristic properties. iPSCs from three CADASIL patients with p.Arg182Cys, p.Arg141Cys and p.Cys106Arg mutations were differentiated into MCs and their functional and molecular profiles were compared. The differentiated CADASIL MCs recapitulated pathogenic changes reported previously: increased PDGFRβ and abnormal structure/distribution of filamentous actin network, as well as N3ECD/LTBP-1/HtrA1-immunopositive deposits. Migration rate of CADASIL MCs was enhanced but suppressed by knockdown of *NOTCH3* or *PDGFRB*. CADASIL MCs showed altered reactivity to PDGF-BB. Patient-derived MCs can recapitulate CADASIL pathology and are therefore useful in understanding the pathogenesis and developing potential treatment strategies.

## Introduction

Cerebral autosomal dominant arteriopathy with subcortical infarcts and leukoencephalopathy (CADASIL) is the most common form of hereditary cerebral small vessel diseases of the brain. The causative gene has been identified as *NOTCH3*, which is specifically expressed in mural cells (MCs) including vascular smooth muscle cells (VSMCs) and pericytes [1, 2]. Over 250 reported *NOTCH3* mutations are reported to be distributed throughout 34 epidermal growth factor-like repeats in the NOTCH3 extracellular domain (N3ECD), all resulting in similar phenotypes such as VSMC degeneration, deposition of granular osmiophilic materials (GOM) in the vasculature, thickening of vessel wall, enlarged perivascular spaces and white matter abnormalities [3, 4]. Clinical and animal studies suggest abnormal vascular reactivity and microvascular rarefaction contribute to white matter changes [5–7]. Although many studies have attempted to unravel how *NOTCH3* mutations lead to artery defects, the pathogenesis of CADASIL is still largely unknown. CADASIL-like rat *NOTCH3* mutations p.Arg171Cys, p.His184Cys, p.Cys544Tyr and p.Arg560Cys, for example, were reported to produce mutant receptors but without any abnormalities in processing, maturation and ligand interaction [8]. Another mutation, p.Arg141Cys, impaired S1 cleavage and thus reduced resultant mature heterodimeric mutant receptors on the cell surface, though signaling activity itself was intact [9]. On the other hand, mutations in the ligand-binding domain (p.Cys428Ser) could result in ligand-binding defects and reduced transcriptional activity [10, 11]. Thus far, there is no clear consensus on the involvement of canonical Notch3 signaling pathway in the pathogenesis of CADASIL, though recent studies seem to support gain of toxic function rather than loss of function [5, 12, 13].

Here, we generated induced pluripotent stem cells (iPSCs) from skin biopsy samples of three CADASIL patients with mutations in the mutational hot spots, exons 2–4 of *NOTCH3,* and differentiated them into MCs to establish in vitro model for elucidating the pathogenesis of CADASIL.

## Materials and methods

All the experiments were repeated at least three times to confirm reproducibility.

### Study subjects and iPSCs generation

Three CADASIL patients with confirmed mutations (CAD1, p.Arg182Cys; CAD2, p.Arg141Cys; and CAD5, p.Cys106Arg) in the *NOTCH3* gene were recruited for this study. Skin biopsy or venipuncture was conducted following Institutional Review Board approval and written informed consent. Human iPSCs were generated by retroviral or episomal transduction of human cDNAs (CAD1: pMXs-hOCT3/4, pMXs-hSOX2, pMXs-hKLF4, pMXs-hc-MYC; CAD2: pCXLE-hOCT3/4-shp53-F, pCXLE-hSK; CAD5: pCXLE-hOct3/4-shp53-F, pCXLE-hSK, pCXLE-hUL, pCXWB-EBNA1) as reprogramming factors in isolated human skin fibroblasts (CAD1, CAD2) or human peripheral blood mononuclear cells (CAD5) [14, 15]. CADASIL iPSCs were confirmed to have normal karyotypes and pluripotency to differentiate into all three germ layers (Additional file 1: Figure S1A–C). Four previously established iPSC clones (N117, TIG107, TIG114 and TIG120) without neurodegenerative or cerebrovascular diseases were selected as controls. All control iPSCs were genetically screened and confirmed not to carry the *NOTCH3* mutation. iPSCs were maintained on SNL feeder layers in Primate ES Cell Medium (ReproCELL) supplemented with 4 ng/ml basic FGF (Peprotech) at 37 °C, 5% CO_2_ and 90–95% humidity.

### Differentiation of iPSCs into MCs

**Fig. 1:**
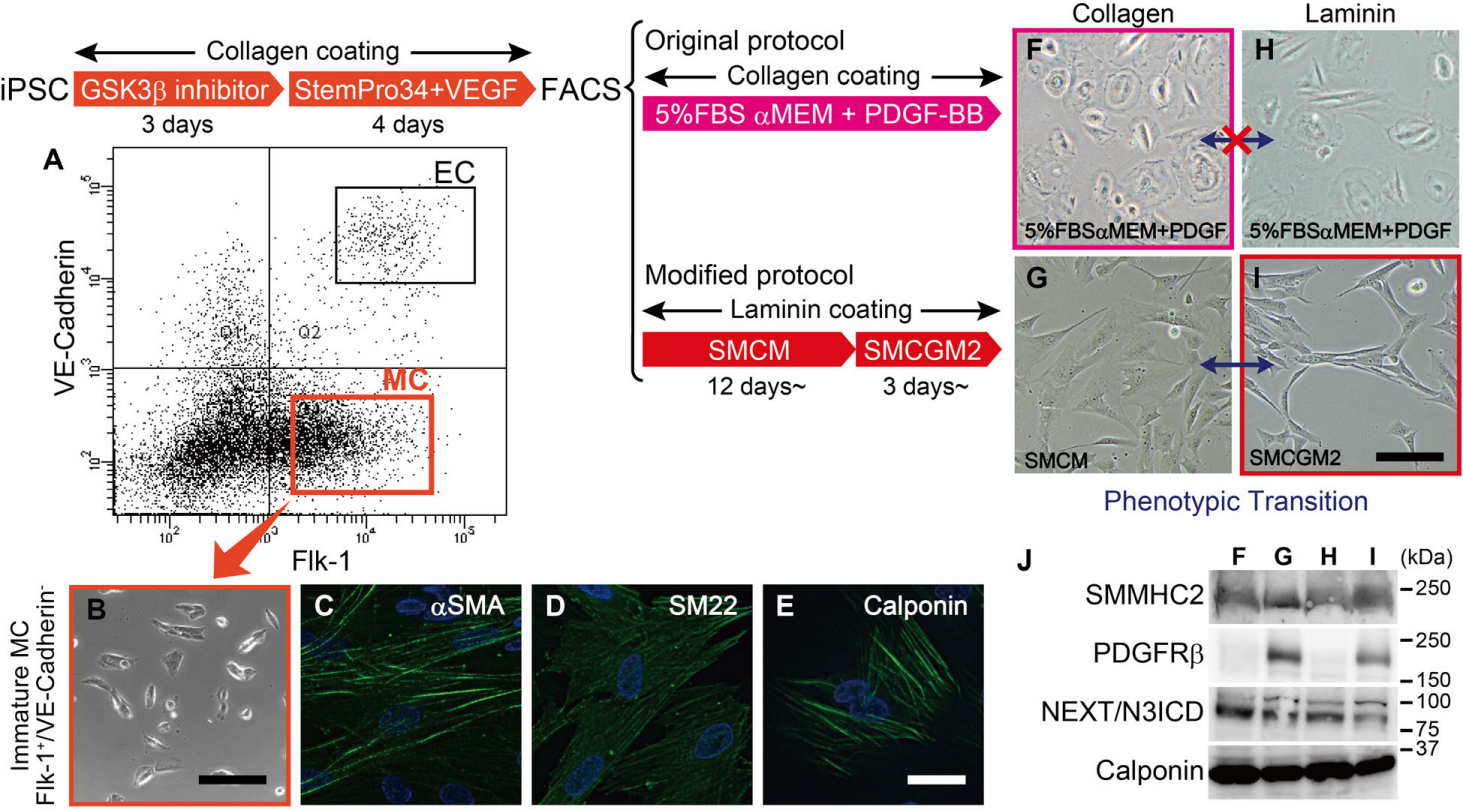
Differentiation of iPSC derived MCs. iPSCs were treated by GSK3β inhibitor, then StemPro34 and VEGF165, to obtain Flk-1 positive/VE-Cadherin negative mural cells (MCs, **a**). Differentiated mural cells were immature at the time of the sorting (**b**) and further cultured for maturation. MCs expressed VSMC marker αSMA, SM22 and calponin (**c**-**e**). Endothelial cells (ECs) can be also isolated as Flk-1 positive/VE-Cadherin positive cells. MCs differentiated by modified protocol changed their morphology depending on the culture condition (**g** and **i**, phenotypic transition), but not MCs differentiated by original protocol (**f** and **h**). Control MC (TIG107) differentiated in αMEM supplemented with 5%FBS and PDGF-BB showed markedly low PDGFRβ, irrespective of the coating (**j**, lane F and H). Bars represent 20 μm in (**c**-**e**), 150 μm in (**b**, **f**-**i**)

iPSCs were differentiated into MCs by a slight modification of a previously described method (Fig. 1) [16, 17]. Briefly, iPSCs were suspended in WiCell conditioned medium (bFGF-, 20%KSR, 1 mM L-Glutamine, 0.07% 2-mercaptoethanol and non-essential amino acid in DMEM/F12) and seeded onto collagen-I coated dishes. The next day, the cells were cultured in WiCell conditioned medium supplemented with 5 μM BIO ((2’Z,3′E)-6-Bromoindirubin-3′-oxime, Sigma-Aldrich, #B1686) and B27/N2 (Thermo Fisher) for 3 days, followed by StemPro-34 (Thermo Fisher) supplemented with VEGF165 (50 ng/ml, Peprotech) for 4 days. MCs were isolated as TRA-1-60-negative, CD144-negative, and flk1-positive cells from the resultant mixture of differentiated cells using a flow cytometer (FACS aria II, BD Biosciences). Immature MCs were further cultured either on collagen I-coated dish in αMEM supplemented with 5% FBS and 10 ng/ml PDGF-BB, or on laminin-coated dish in Smooth Muscle Cell Medium (SMCM, ScienCell) for 12 days before cryopreservation. All clones differentiated in SMCM were thawed simultaneously and cultivated in SMCM, and then in Smooth Muscle Cell Growth Medium 2 (SMCGM2, PromoCell) for more than 3 days before the start of each experiment.

### Immunofluorescence

iPSC-derived MCs were cultured in μ-slide VI 0.4 (ibidi) and fixed in 4% paraformaldehyde (PFA) and permeabilized with 0.1% TritonX-100. After blocking with 1% BSA/PBS, cells were immunostained with primary antibodies. The following antibodies were used in this study: anti-αSMA, anti-SM22, anti-GRP78 BiP and anti-58 K Golgi protein antibodies from Abcam, anti-calponin antibody from Dako, anti-smooth muscle myosin heavy chain 2 (SMMHC2) antibody from Yamasa (Japan), anti-human latent TGF-β binding protein-1 (LTBP-1) antibody and anti-high temperature requirement A1 (HTRA1) antibody from R&D systems, anti-LAMP1 antibody from Santa Cruz Biotechnology, and anti-PDGFRβ from Cell Signaling Technology. The antibody against human N3ECD was kindly provided by Dr. Atsushi Watanabe (National Center for Geriatrics and Gerontology, Japan) [18, 19]. Alexa Fluor 488 or 546 conjugated donkey anti-Rabbit/Mouse IgG (Thermo Fisher) antibodies were used as secondary antibodies. For live cell staining, anti-PDGFRβ antibody was diluted in SMCGM2, and the MCs incubated at 37 °C for 1 h. After rinsing with PBS, MCs were fixed in 4% PFA and incubated in secondary antibody conjugated with AlexaFluor 488. Filamentous actin (F-actin) was stained using either Alexa Fluor 488 Phalloidin (Cell Signaling Technology) or Acti-stain 555 fluorescent phalloidin (Cytoskeleton). Images were taken using KEYENCE BZ-9000 and BZ-X800. ImageJ software was used to quantify integrated density of N3ECD staining per cell.

### Western blot

MCs were lysed in lysis solution (1% SDS, 4 M urea, 1 mM EDTA, 150 mM NaCl, 50 mM Tris, protease inhibitor cocktail (nacalai tesque), pH 8.0) and ultrasonic treatment applied. For western blotting the following antibodies were used: SMMHC2 and GAPDH from abcam, Notch1, Notch2, Notch3 and HES1 from Cell Signaling Technology, Calponin from Millipore, Caldesmon from Santa Cruz Biotechnology, peroxidase-conjugated secondary antibodies from Jackson ImmunoResearch.

### Proliferation assay

MCs were seeded in 96-well plates at 5 × 10^3^/well in triplicate and incubated overnight. Cell number was measured the next day and every subsequent day for up to 4 days using Cell Counting Kit-8 (Dojindo Molecular Technologies). Tetrazolium salt WST-8 was added to the medium and the cells incubated at 37 °C for 1 h before measuring OD at 450 nm. For the evaluation of PDGF-BB reactivity, MCs were seeded and incubated overnight before being serum deprived in OPTI-MEM (Thermo Fisher Scientific) for 6 h. The MCs were then treated with PDGF-BB (Peprotech) in various concentration ranges from 0 to 30 ng/mL for 2 days. WST-8 was added to each well and incubated at 37 °C for 1.5 h.

### Collagen gel contraction assay

Collagen gel contraction assays were conducted in triplicate, as previously described with slight modification [20]. MCs were first treated with mitomycin C to eliminate the influence of cell proliferation. The cells were then suspended in smooth muscle cell basal medium 2 (SMCBM2, PromoCell) and mixed with type I collagen at a final concentration of 2.5 × 10^5^ cells/ml and 2 mg collagen/ml. Each mixture was immediately dispensed into a 24-well plate and incubated at 37 °C for 1 h. The gels were solidified and 10% FBS-SMCBM2 gently added and the gels carefully detached from the walls of the well using a spatula. The gels were further incubated for 24 h and images taken to measure the areas of gels using NIH ImageJ software. All values were normalized and presented in percentage of the measured well area.

### Migration assay

MCs were seeded into Culture-Insert 2 Well (ibidi) at 2.6 × 10^4^/well. After overnight incubation, the insert was removed, and images taken immediately and every 3 h for up to 12 h thereafter. The images were then analyzed using automated analysis software WimScratch (Wimasis). Data was presented as percentage of cell-covered area to the initial wound area at 0 h.

### Distribution of F-actin and G−/F-actin ratio

MCs were seeded into μ-Slide VI flow through (ibidi) and fixed in 4% paraformaldehyde. Cells were stained with Alexa Fluor 488 Phalloidin and embedded in SlowFade Diamond Antifade Mountant with DAPI (Life Technologies). The ratio of globular/filamentous-actin (G/F-actin) was quantified using G-Actin/F-Actin In Vivo Assay Biochem Kit (Cytoskeleton).

### Manipulation of notch signaling pathway

Transfection of small interfering RNA (siRNA) was carried out using Lipofectamine 3000 (Thermo Fisher Scientific). Suspension of MCs were treated with 50 nM Universal Negative Control siRNA or human *NOTCH3* siRNA (MISSION siRNA, SIC-001 and SASI_Hs02_00302544, all from Sigma Aldrich), and seeded into laminin-coated 6-well plates. MCs were incubated in SMCGM2 and used for experiments on day 3. MCs were also treated with γ-secretase inhibitor DAPT (N-[N-(3,5-difluorophenacetyl)-l-alanyl]-S-phenylglycine t-butyl ester, Calbiochem) at the concentration of 25 μM for 24 h.

### Statistical analysis

Values are presented as mean + standard error of the mean (SEM). Statistical significance was evaluated using t-test to compare between two groups and one-way ANOVA followed by Tukey’s post hoc test for multiple comparisons. Time course difference in proliferation and migration rate was analyzed using a general linear model with repeated measurements. *P* < 0.05 was considered statistically significant. *P* values were adjusted using Benjamini-Hochberg procedure for multiple testing.

## Results

### Differentiation of iPSC into MCs

iPSCs were differentiated into MCs by a previously described method with slight modifications [16, 17]. Differentiated cells were sorted by FACS, and Flk-1-positive and VE-cadherin-negative cells were isolated as MCs (Fig. 1a). The iPSC-derived MCs were immature at the time of sorting (Fig. 1b) and thus cultured either on collagen I-coated dish in 5%FBS, 10 ng/ml PDGF-BB in αMEM (original protocol), or on laminin-coated dish in SMCM, then SMCGM2 (modified protocol), for maturation before the property of the cells were validated. Immunocytochemistry revealed the MCs expressed VSMC markers including αSMA, SM22 and calponin (Fig. 1c-e). The morphology was heterogeneous in MCs differentiated by the original protocol, with mixture of flat and round cells and spindle-shaped cells (Fig. 1f), while most cells are spindle-shaped and relatively homogeneous when differentiated by the modified protocol (Fig. 1i). MCs differentiated on laminin-coated dish in SMCM changed their morphology and cellular properties depending on the culture conditions: flat and spread-shaped MCs cultured in SMCM on collagen I-coated dish transformed to elongated spindle-shaped cells, forming a network-like structure, when cultured in SMCGM2 on a laminin-coated dish (Fig. 1g and i). Such reversible shifts between distinct morphological and functional properties (phenotypic transition) is one of the features of VSMCs and was not observed in MCs differentiated by the original protocol (Fig. 1f and h). The difference in the composition of culture media also affected the expression of PDGFRβ (Fig. 1j, lane F and H vs. G and I) but not NOTCH3, SMMHC2 and calponin.

### Recapitulation of CADASIL phenotypes

**Fig. 2:**
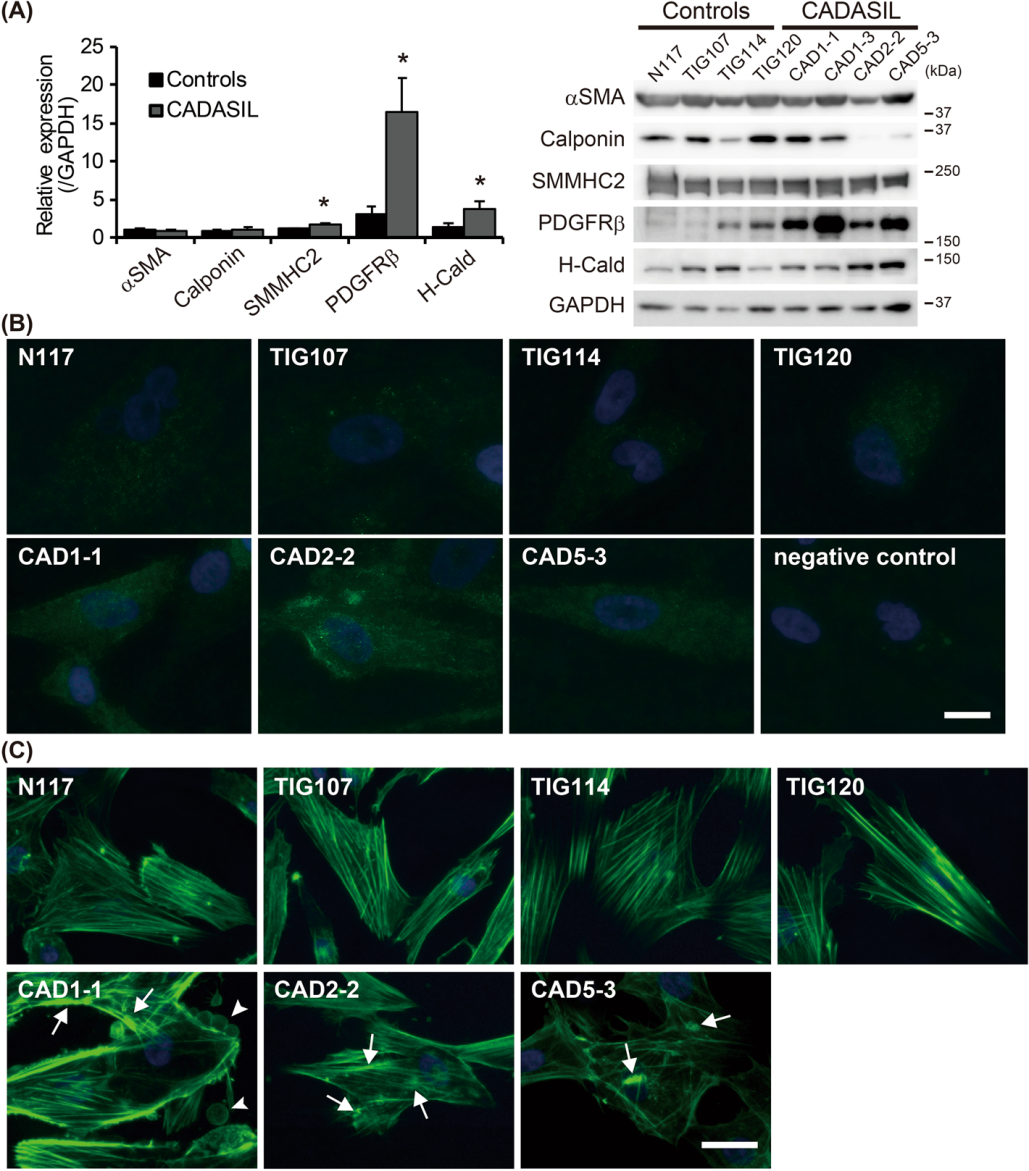
Recapitulation of CADASIL phenotypes. **a** Western blotting analysis of VSMC markers revealed significantly upregulated PDGFRβ, SMMHC2 and H-caldesmon (H-Cald) in CADASIL MCs (*n* = 3) compared to control (*n* = 4). **b** shows representative images of the increased PDGFRβ in CADASIL. **c** Actin cytoskeleton in CADASIL MCs was often irregular and unevenly distributed, forming aggregation, or nodes (arrows). Occasionally, the cells showed a ‘bleb’-like structure on the surface (arrowheads), which was rarely present in controls. Bars represent 10 μm in (**b**) and 25 μm in (**c**). The error bars represent the SEM. *p < 0.05

We then evaluated whether iPSC-derived MCs cultured in SMCM/SMCGM2 recapitulate the CADASIL phenotype reported previously, i.e. abnormal actin cytoskeleton, N3ECD accumulation and increased platelet-derived growth factor receptor β (PDGFRβ) [18, 21–24]. The differentiated MCs expressed various VSMC markers, e.g. αSMA, calponin, SMMHC2, PDGFRβ and H-caldesmon. In particular, PDGFRβ was dramatically increased in CADASIL MCs compared to controls (Fig. 2a and b, *p* = 0.012). CADASIL MCs also showed significant increase in SMMHC2 and H-caldesmon levels (*p* = 0.019 and 0.040), which are proteins related to contraction.

**Fig. 3:**
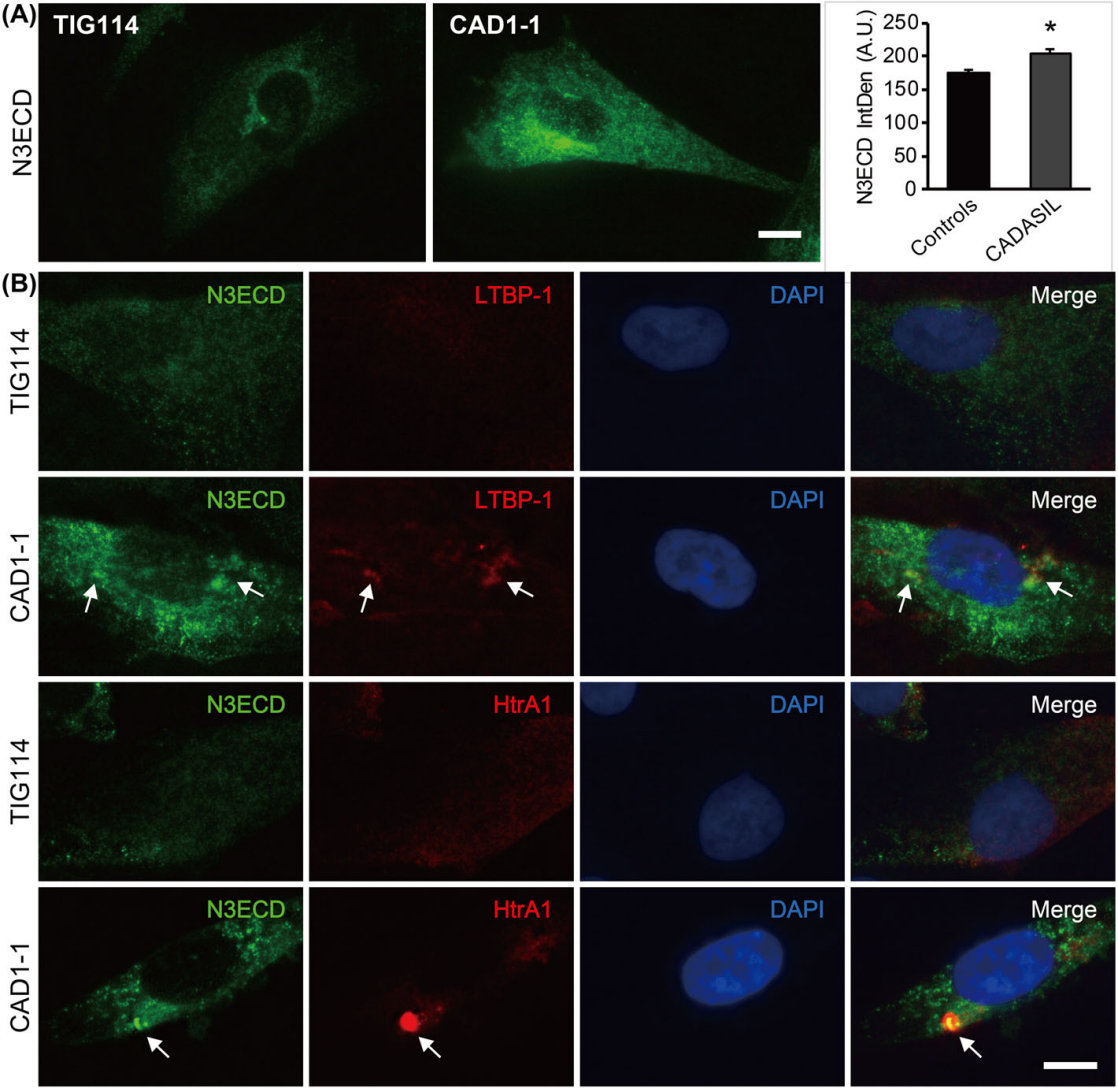
Immunoreactivity of components of granular osmiophilic materials. **a** Representative images of N3ECD staining. Integrated density (IntDen) of N3ECD immunoreactivity (IR) was significantly increased in CADASIL MCs (*n* = 3) than controls (*n* = 4). **b** Some of N3ECD deposits in CADASIL MCs were positive for LTBP-1 and HtrA1 (arrows). Bars represent 10 μm. *** *p* < 0.0001

Phalloidin staining revealed increased branching of F-actin, formation of nodes (Fig. 2c, arrows) and an uneven, irregular distribution of F-actin bundles in CADASIL as previously reported [22]. In addition, CADASIL MCs often showed “bleb”-like structures on the cell surface (Fig. 2c, CAD1–1, arrowheads), while there was no sign of apoptosis, such as cell shrinkage and nuclear fragmentation. The bleb-like structure in CADASIL MCs contained both N3ECD and NOTCH3 intracellular domain (N3ICD) at an intensity similar to that in the cytoplasm (Additional file 2: Figure S2). Detailed examination of N3ECD immunoreactivity revealed significantly intense (*p =* 0.021) and larger aggregate-like staining unevenly distributed in CADASIL MCs, sometimes within the Golgi apparatus and not in endoplasmic reticulum, while it was often uniformly distributed throughout the cell in controls (Fig. 3a and Additional file 3: Figure S3A and B). N3ECD-positive deposits sized 1–2 μm were occasionally present on the plasma membrane of CADASIL MCs, some of which were positive for reported components of GOM, latent-transforming growth factor beta-binding protein-1 (LTBP-1) and high temperature requirement A1 (HtrA1) (Fig.3b, Additional files 4 and 5: Figure S4A and Figure S5A, arrows). N3ICD, however, did not colocalize with LTBP-1 and HtrA1 (Additional files 4 and 5: Figure S4B and Figure S5B). It is of note that strong LTBP-1 immunoreactivity did not consistently colocalize with mutant N3ECD, with many positive for either LTBP-1 or N3ECD only (Additional file 4: Figure S4A, arrowheads), while most strong HTRA1 immunoreactivity was positive for mutant N3ECD (Additional file 5: Figure S5A). These data suggested that iPSC-derived MCs were sufficient as an in-vitro model of CADASIL.

### Altered migration in CADASIL

**Fig. 4:**
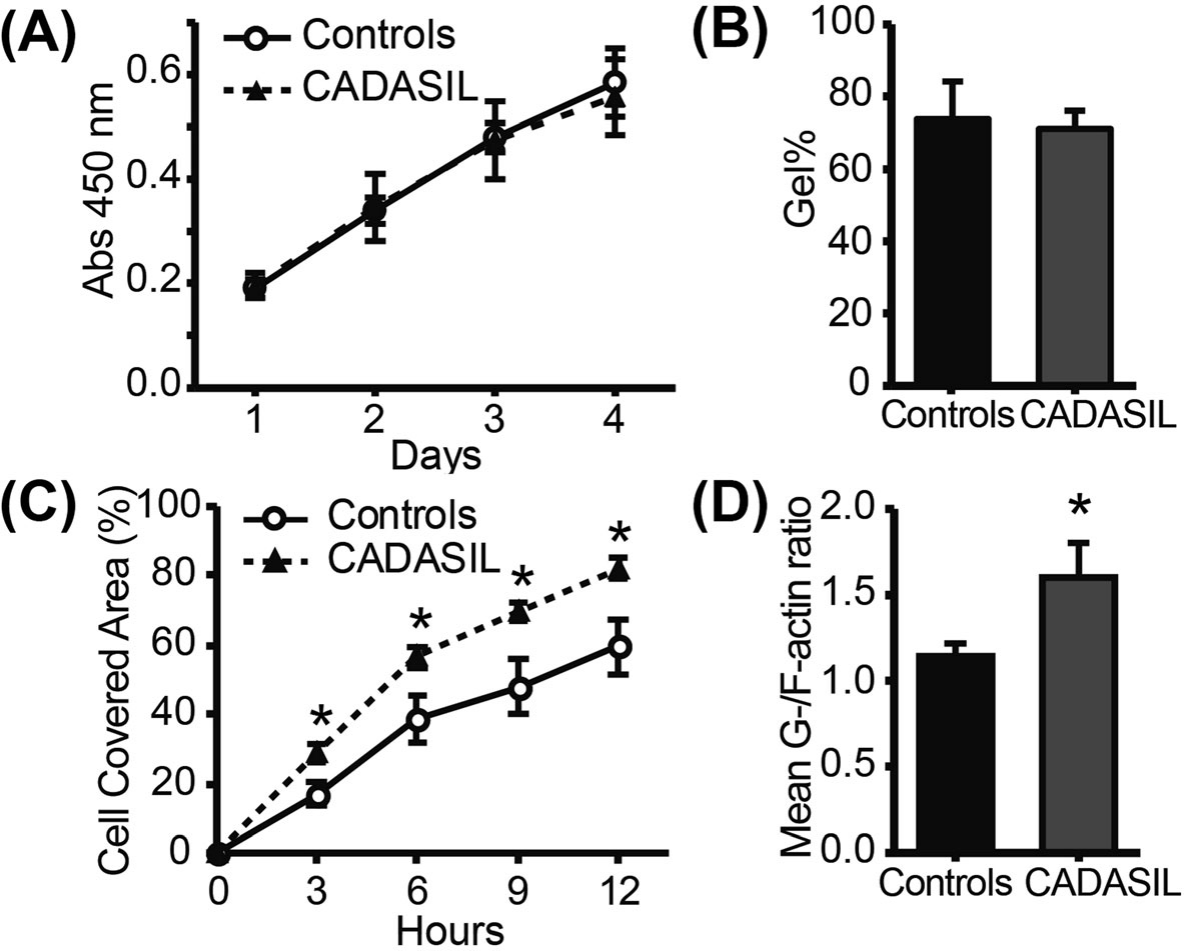
Functional differences of CADASIL MCs (*n* = 3) compared to controls (*n* = 4). **a** The proliferation was assessed by a WST-8 assay daily for 4 days; no significant difference was found. **b** Collagen contraction assay showed no significant difference in contraction between controls and CADASIL. **c** Cell migration, as evaluated by the scratch assay, was significantly increased in CADASIL iPSMCs, which was also evident from the activated actin metabolism as indicated by the increased G−/F- actin ratio (**d**). The error bars represent the SEM. *p < 0.05

To elucidate how the *NOTCH3* mutations affect cellular functions, proliferation, contraction and migration assays were conducted. The proliferation rate greatly varied between samples rather than between groups; thus, overall, there was no significant difference between controls and CADASIL (Fig. 4a). Despite the significant increase in contraction-related proteins (Fig. 2a), cell contractility, indicated by shrinkage of the cell-containing collagen disk cultured in 10% FBS/SMCGM2, was not affected by *NOTCH3* mutations (Fig. 4b). The only significant difference was observed in migration rate, which was evaluated by a wound healing assay (Fig. 4c). CADASIL MCs migrated significantly faster toward the wound area than controls (cell covered area: 69.5 ± 2.49% vs. 47.9 ± 7.92% after 9 h, *p* = 0.012). Migration is mediated by the polymerization and depolymerization of actin. The ratio of G-actin to F-actin was indeed significantly increased in CADASIL (*p* = 0.037; Fig. 4d).

### Knockdown of *NOTCH3* and *PDGFRB* attenuated stimulated migration speed in CADASIL

**Fig. 5:**
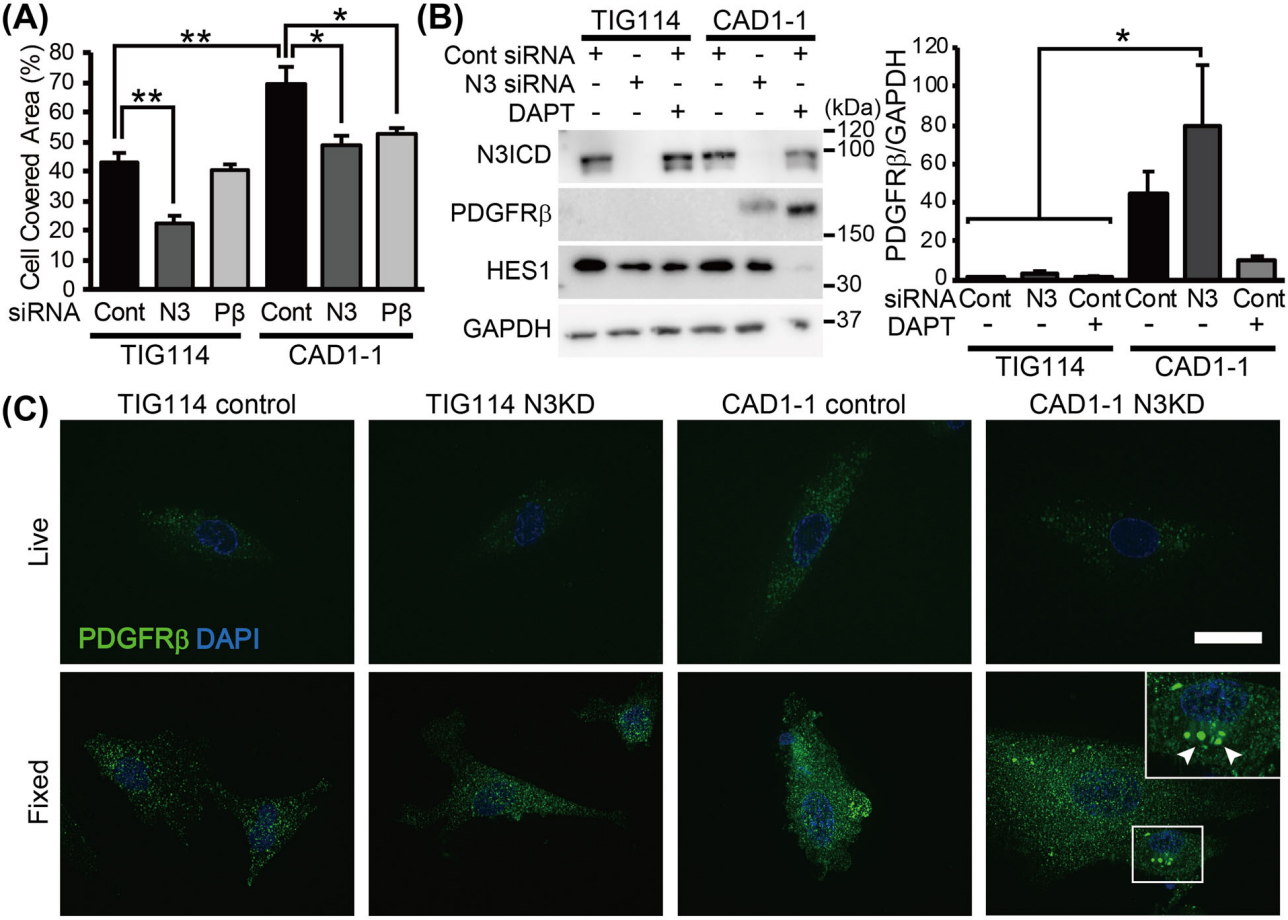
Relationships between NOTCH3, PDGFRβ and migration. **a** Cell covered area was measured at 9 h after the start of migration assay. Both *NOTCH3* and *PDGFRB* knockdown resulted in the reduced migration rate in CADASIL iPSMCs (CAD1–1 N3 and Pβ) to the control level (TIG114 Cont). However, *NOTCH3* knockdown resulted in further increase of PDGFRβ in CADASIL, while inhibition of DAPT treatment significantly reduced expression (**b**). **c** Immunofluorescent staining of PDGFRβ revealed increased surface and cytoplasmic PDGFRβ in CADASIL MCs (CAD1–1 control, Live and Fixed). Further increase of PDGFRβ by *NOTCH3* siRNA transfection (N3KD) was explained by the intracellular aggregation of excessive PDGFRβ (arrowheads) and reduced functional receptors on the plasma membrane (CAD1–1 N3KD, fixed). Bar represents 20 μm. The error bars represent the SEM. *p < 0.05, **p < 0.01

The relationships between *NOTCH3* mutation and increased migration rate in CADASIL MCs were investigated using MCs of a representative case from each group (control, TIG114; CADASIL, CAD1), which showed average migration rate within each group. The MCs were transfected with control siRNA or *NOTCH3*/*PDGFRB* siRNA 3 days before the start of wound healing assay. Both *NOTCH3* and *PDGFRB* knockdown significantly reduced migration rate in CADASIL to the control level (TIG114 control siRNA vs. CAD1 control siRNA, *p* = 0.001; CAD1 control siRNA vs. CAD1 *NOTCH3* siRNA, *p* = 0.007; CAD1 control siRNA vs. CAD1 *PDGFRB* siRNA, *p* = 0.043; TIG114 control siRNA vs. CAD1 *NOTCH3* siRNA, *p* = 0.848; TIG114 control siRNA vs. CAD1 *PDGFRB* siRNA, *p* = 0.370; Fig. 5a). Since *NOTCH3* knockdown did not stimulate cellular migration in controls and both *NOTCH3* and *PDGFRB* knockdown in CAD1 resulted in the normalized migration rate, we hypothesized that CADASIL is caused by a gain of toxic function of mutant NOTCH3 through excessive PDGFRβ. The amount of N3ICD and HES1 varied depending on the cell condition at the time of sampling and no consistent difference was found between control and CADASIL MCs (Fig. 5b, lane 1 and 4 from left, respectively; Additional file 6: Figure S6A). Unexpectedly, *NOTCH3* knockdown resulted in a further increase of PDGFRβ in CAD1 MCs, while treatment with a γ-secretase inhibitor (DAPT) significantly decreased the expression (Fig. 5b, lane 5 and 6). Same trend was observed in CAD5, and longer (~ 7 days) *NOTCH3* knockdown period also resulted in the increased PDGFRβ (Additional file 6: Figure S6B). In addition, *NOTCH3* knockdown did not significantly alter downstream HES1 level. To elucidate the cause of the conflicting consequences of PDGFRβ level and migration rate by *NOTCH3* knockdown and DAPT treatment, the distribution of PDGFRβ reactivity was determined by immunofluorescent staining (Fig. 5c). siRNA-transfected MCs were incubated with anti-PDGFRβ antibody either before (Live) or after fixation (Fixed). CADASIL MCs showed more PDGFRβ both on the plasma membrane (upper third panel) and within a whole cell (lower third panel) compared to controls (far-left panels). The excessive PDGFRβ in *NOTCH3* siRNA-transfected CADASIL MCs formed intracellular deposits (inset in the lower far-right panel, arrowheads), which may have consequently reduced the reactive receptors on the plasma membrane (upper far-right panel) and thus suppressed migration rate. Similar aggregates were occasionally observed in control siRNA-transfected CADASIL MCs, but not as frequently as in *NOTCH3* knocked-down CADASIL MCs. The aggregates were not found to be labelled by markers of the endoplasmic reticulum (GRP78), Golgi apparatus (58 K Golgi protein) or lysosomes (LAMP1) (data not shown).

### Increased PDGFRβ alters proliferation response to PDGF-BB in MCs

**Fig. 6:**
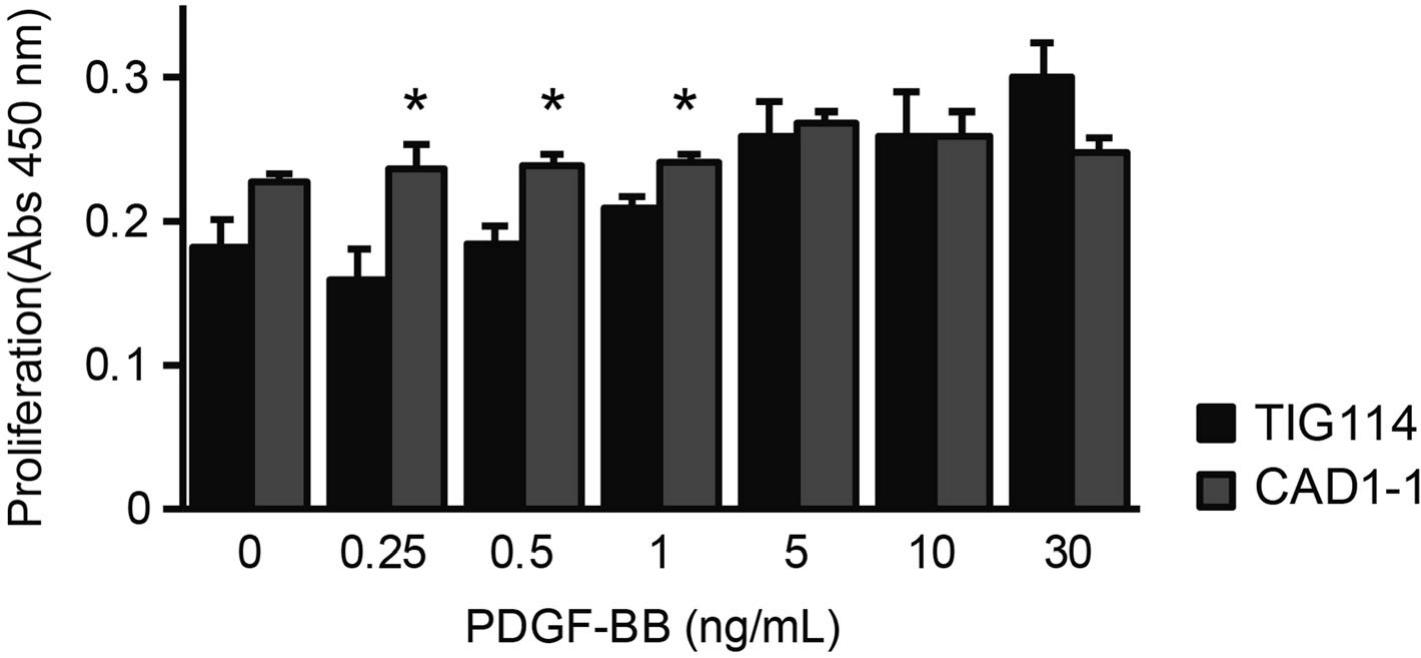
PDGF-BB-induced proliferation of MCs. CADASIL (CAD1–1) MCs started to proliferate at the lower PDGF-BB concentrations than control (TIG114) MCs, suggesting an altered response to PDGF. The error bars represent the SEM

PDGF are known to regulate both cell migration and proliferation: lower PDGF concentration induces migration whereas higher concentration (> 5 ng/ml) promotes proliferation [25]. To elucidate the effect of increased PDGFRβ and its involvement in CADASIL pathogenesis, we cultured control and CADASIL MCs in serum-free medium with increasing concentration of PDGF-BB and compared their proliferation rate (Fig. 6). CADASIL MCs proliferated significantly more than controls even at lower concentration (PDGF-BB 0.25 ng/ml, *p* = 0.015; 0.5 ng/ml, *p* = 0.009; 1.0 ng/ml, *p* = 0.015), likely due to increased PDGFRβ.

## Discussion

Here, we present our CADASIL iPSC-derived MCs as an in vitro model of CADASIL. The primary aim of the current study was to establish a differentiation protocol for MCs that can recapitulate the phenotypes of CADASIL reported in the literature. We demonstrated that three CADASIL iPSCs with point mutations in exon 3–4 were successfully differentiated into MCs, which showed abnormal actin cytoskeleton as well as increased PDGFRβ, as previously reported in studies using primary cell culture and post-mortem brain tissues [21, 22]. The increased PDGFRβ expression in the iPSC-derived MC explained why PDGFRβ immunoreactivity was increased in the postmortem brains of CADASIL patients despite the degeneration of MCs in the arterioles and capillaries [21]. Our data on F-actin aggregation in MCs from three CADASIL patients (p.Arg182Cys, p.Arg141Cys, p.Cys106Arg) is also consistent with the recent report of iPSC-derived VSMCs from a single patient with p.Arg1076Cys mutation, suggesting that the mutations in exon 3/4 and exon 20 result in the same outcome, at least in terms of actin abnormality [26]. The advantage of our CADASIL MCs is that our MCs could, for the first time, recapitulate N3ECD deposition colocalized with LTBP-1 and HtrA1, which are reported as components of GOM [18, 23, 24]. These N3ECD depositions were relatively rare and found on one in a few dozens of cells, possibly because they are dispersed into the culture media over time. Interestingly, the strong LTBP-1 immunoreactivity did not always colocalize with mutant N3ECD, while most of HTRA1 did, confirming the report of Zellner et al., which showed colocalization of HTRA1 and N3ECD deposits in patient vessels [24]. Mutations in *HTRA1* is known to cause CARASIL, a recessive form of hereditary small vessel disease [27]. Although phenotypes of CADASIL and CARASIL do not fully overlap each other [28], the colocalization of N3ECD and HTRA1 implies common underlying mechanism leading to the vascular defect. Overall, these data suggest iPSC-derived MCs are a useful tool as an in vitro model of CADASIL pathogenesis.

Further investigation on the functional differences has revealed *NOTCH3* mutations did not affect cell proliferation and contraction, but significantly accelerated migration in CADASIL MCs. The increased migration rate was suppressed by the knockdown of *NOTCH3* and *PDGFRB*. PDGFRβ is a receptor tyrosine kinase that is involved in cellular migration and proliferation [29], and has been reported to be upregulated by Notch1 and Notch3 in VSMCs [30]. It is of note that knockdown of *NOTCH3* in control MCs did not result in the significant upregulation of PDGFRβ or increased migration, as observed in control siRNA-treated CADASIL MCs, discounting loss of function as a pathogenic mechanism.

Decreased PDGFRβ after treatment with γ-secretase inhibitor DAPT indicated a close relationship between Notch signaling pathways and PDGFRβ-mediated migration. Indeed, several studies have reported that Notch1 and 3 are involved in the regulation of PDGF signaling pathway [30–32]. Notch1 ICD binds to a proximal promoter region of PDGFRβ, while N3ICD is recruited to another CSL-binding site outside the promoter region to activate the transcription of PDGFRβ [30]. Decreased migration rate by DAPT treatment and *NOTCH3* knockdown supported the regulatory mechanism and appeared to suggest CADASIL pathology is caused by the increased PDGFRβ by gain of function *NOTCH3* mutations. However, the increased PDGFRβ by mutant *NOTCH3* knockdown confounded expectations from previous studies [30, 32], and appeared to be dominant negative effect. The decreased migration rate could be explained by the intracellular aggregation of excessive PDGFRβ, despite the increased amount of PDGFRβ, resulting in reduced reactive receptors on the plasma membrane. One plausible explanation is that our study performed knockdown of *NOTCH3*, rather than knockout as in previous reports [30, 32]. Gene knockout, but not knockdown by siRNA, is known to trigger transcriptional adaptation, which upregulates genes that exhibit sequence similarity to the degraded mRNA, often those belonging to the same family [33]. While canonical NOTCH1 signaling pathway is competitively inhibited by N3ICD, both N1ICD and N3ICD directly induce PDGFRβ expression [34–36]. The presence or absence of transcriptional adaptation could have affected the PDGFRβ expression. Conversely, the increased PDGFRβ may be a result of compensation by other Notch signaling pathways, independent of HES1 [30], which were inhibited by non-selective γ-secretase inhibitor DAPT treatment but not by *NOTCH3* knockdown. Another possibility is that our experiment was conducted without any additional ligand stimulation, which could have affected the complex regulatory mechanisms behind Notch signaling by cis-inhibition and/or trans-activation. Stimulation with ligands, such as Jagged1 may elicit a different outcome.

The pathological mechanism of CADASIL has long been of interest for researchers investigating small vessel disease of the brain. The current study showed that increased PDGFRβ in CADASIL MCs resulted in cellular proliferation at significantly lower PDGF-BB concentration. However, a recent study by Kelleher et al. [37] reported decreased PDGFRβ in CADASIL iPSC (p.Arg153Cys and p.Cys224Tyr)-derived MCs. Although our study used CADASIL iPSCs with different *NOTCH3* mutations from theirs, they are all located in exon3/4. Thus, the opposing results on PDGFRβ expression would rather stem from the difference in the differentiation protocols. PDGF-BB is a possible responsible component, as our data comparing original and modified protocol clearly demonstrated that supplementation of PDGF-BB suppressed the expression of PDGFRβ (Fig. 1j). The fact that morphology of MCs in Kelleher’s study was similar to that of MCs differentiated by original protocol may support the possibility. Considering that our MCs express H-caldesmon, calponin and SMMHC2, markers of VSMC maturation [38], they are probably closer to VSMC while Kelleher’s MCs were pericyte-like, and that could have contributed to the difference in PDGFRβ expression. In either case, increased or decreased, the significant difference itself may matter. During angiogenesis, migration of MC along newly-formed endothelial tubes is controlled by the concentration of PDGF: cells migrate when PDGF concentration is low, and proliferate once cells reach areas with high concentration, meaning even a slight change in the signaling can affect vascular stabilization by MCs [25]. Either increased or decreased, the altered expression of PDGFRβ inevitably affects reactivity to PDGF in CADASIL MCs and thus results in the impaired stabilization of new blood vessels, making them susceptible to mechanical stress.

Limitations of our study are primarily related to the inter-individual variability, even within the same group. The differentiation efficacy of MCs, for example, ranged from 3 to 18%, irrespective of the genotype. We also cannot exclude the possibility that the relatively large standard deviation masked the differences in proliferation and contraction. No clear genotype-phenotype correlation has been reported in CADASIL so far, but environmental factors including vascular risk factors have been suggested to influence the disease onset [39], implying either other genetic background or slight difference in the culture condition may have contributed to the large variability. The fact that our MCs did not consistently show activation of NOTCH3 signaling during the repeatability experiments as reported in the previous study using iPSC from a single patient emphasizes the importance of sufficient sample size and repeated experiments [26]. It is not unlikely that such variations in responses within MCs exist in situ explaining the known wide pathogenic variants of the CADASIL phenotype [40]. Future studies are recommended to include at least 4 cases per group or use control iPSC lines transfected with CADASIL-mutation to minimize the effects of genetic background. Nevertheless, our iPSC-derived MCs possess several advantages as an in vitro model of CADASIL. Firstly, CADASIL iPS-derived MCs were shown to recapitulate CADASIL pathology, at least in part. Secondly, both endothelial cells and MCs can be isolated at the sorting step in our differentiation protocol, enabling co-culture experiments. Thirdly, use of iPSC enables constant supply of large number of MCs in the same generation. Lastly, in addition to mature MCs, cells at different differentiation stages can be evaluated. Considering the relative reasonable cost of iPSC cultures and variations in differentiation efficacy, the use of iPSC-derived MCs would prove a useful tool for unraveling the pathogenesis of CADASIL.

## Supplementary information

**Additional file 1: Figure S1.** Validation of iPSCs. The established CADASIL iPSC lines (CAD1–1, CAD2–2 and CAD5–3) showed normal karyotype (A) and expressed pluripotency markers, Oct4, NANOG and SOX2 (B). The iPSCs could differentiated into all three germ layers in vivo (C). Bar represents 500 μm (B) and 100 μm (C).

**Additional file 2: Figure S2.** Representative images of N3ECD and N3ICD immunoreactivity and blebs. The bleb-like structure in CAD1–1 MC contained both N3ECD and N3ICD, but no more than the adjacent cytoplasm. Bar represents 5 μm.

**Additional file 3: Figure S3.** Representative images of N3ECD immunoreactivity. (A) While N3ECD were relatively uniformly distributed throughout the cell in controls (TIG114), more intense and aggregate-like immunoreactivity was unevenly distributed within the cell in CAD1–1, sometimes within the Golgi apparatus (arrows). Some of the intense staining were unassociated with Golgi apparatus marker (arrowheads) (B) Such strong focal immunoreactivity did not colocalize with an endoplasmic reticulum marker, GRP78 (arrowheads). Bars represent 10 μm.

**Additional file 4: Figure S4.** Colocalization of N3ECD and LTBP1 immunoreactivity. (A) Some of strong N3ECD immunoreactivity in CADASIL MCs were also positive for LTBP-1 (arrows) but the others were positive for either N3ECD or LTBP1 only (arrowheads). (B) LTBP1 did not colocalize with N3ICD immunoreactivity (B). Bars represent 10 μm.

**Additional file 5: Figure S5.** Colocalization of N3ECD and HtrA1 immunoreactivity. Intense HtrA1 immunoreactivity colocalized with N3ECD (A) but not with N3ICD (B). Bars represent 10 μm.

**Additional file 6: Figure S6.** Expression of NOTCH3 and PDGFRβ in MCs. (A) The amount of N3ICD varied depending on the cell condition at the time of sampling and no consistent difference was found between control and CADASIL MCs. (B) Increased PDGFRβ was observed even after 7 days of *NOTCH3* knockdown.

## Data Availability

The datasets used and analyzed during the current study are available from the corresponding authors on reasonable request.

## Abbreviations

F-actin: Filamentous actin
G-actin: Globular actin
GOM: Granular osmiophilic material
HtrA1: High temperature requirement A1
iPSC: induced pluripotent stem cell
LTBP-1: Latent-transforming growth factor β-binding protein-1
MC: Mural cell
N3ECD: Notch3 extracellular domain
N3ICD: Notch3 intracellular domain
NEXT: Notch extracellular truncated domain
PDGF-BB: Platelet-derived growth factor-BB
PDGFRβ: Platelet-derived growth factor receptor β
SEM: Standard error of the mean
SMCGM2: Smooth Muscle Cell Growth Medium 2
SMCM: Smooth Muscle Cell Medium
SMMHC2: Smooth muscle myosin heavy chain 2
VSMC: Vascular smooth muscle cell
