# Supplementary figures and images for: Human iPS cell-derived mural cells as an in vitro model of hereditary cerebral small vessel disease
Source: Mol Brain. 2020 Mar 19;13:38. doi: 10.1186/s13041-020-00573-w (PMC7081541; doi:10.1186/s13041-020-00573-w)

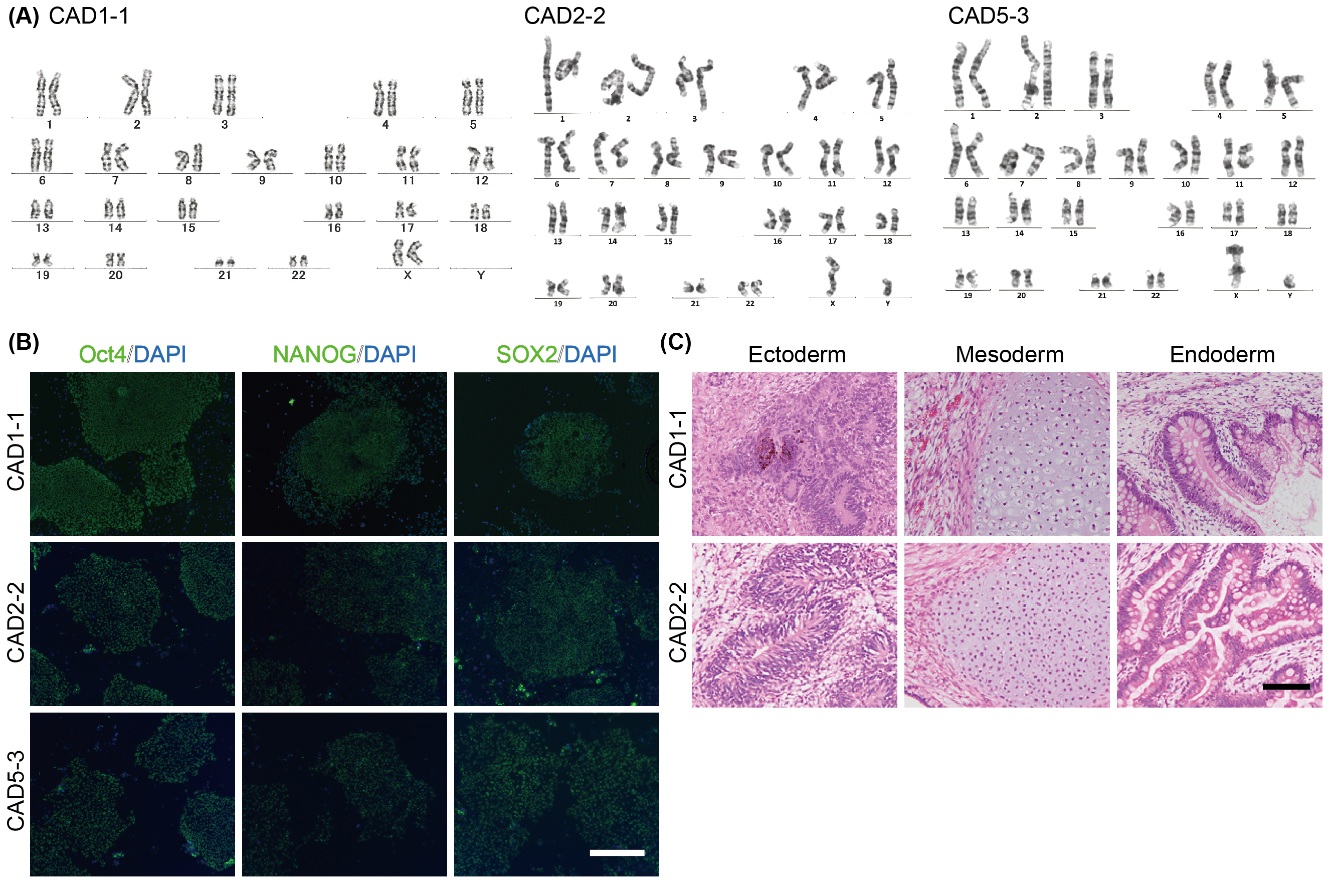

Supplement: Supplementary file 1 — Additional file 1: Figure S1. Validation of iPSCs. The established CADASIL iPSC lines (CAD1–1, CAD2–2 and CAD5–3) showed normal karyotype (A) and expressed pluripotency markers, Oct4, NANOG and SOX2 (B). The iPSCs could differentiated into all three germ layers in vivo (C). Bar represents 500 μm (B) and 100 μm (C). [file 13041_2020_573_MOESM1_ESM.jpg]

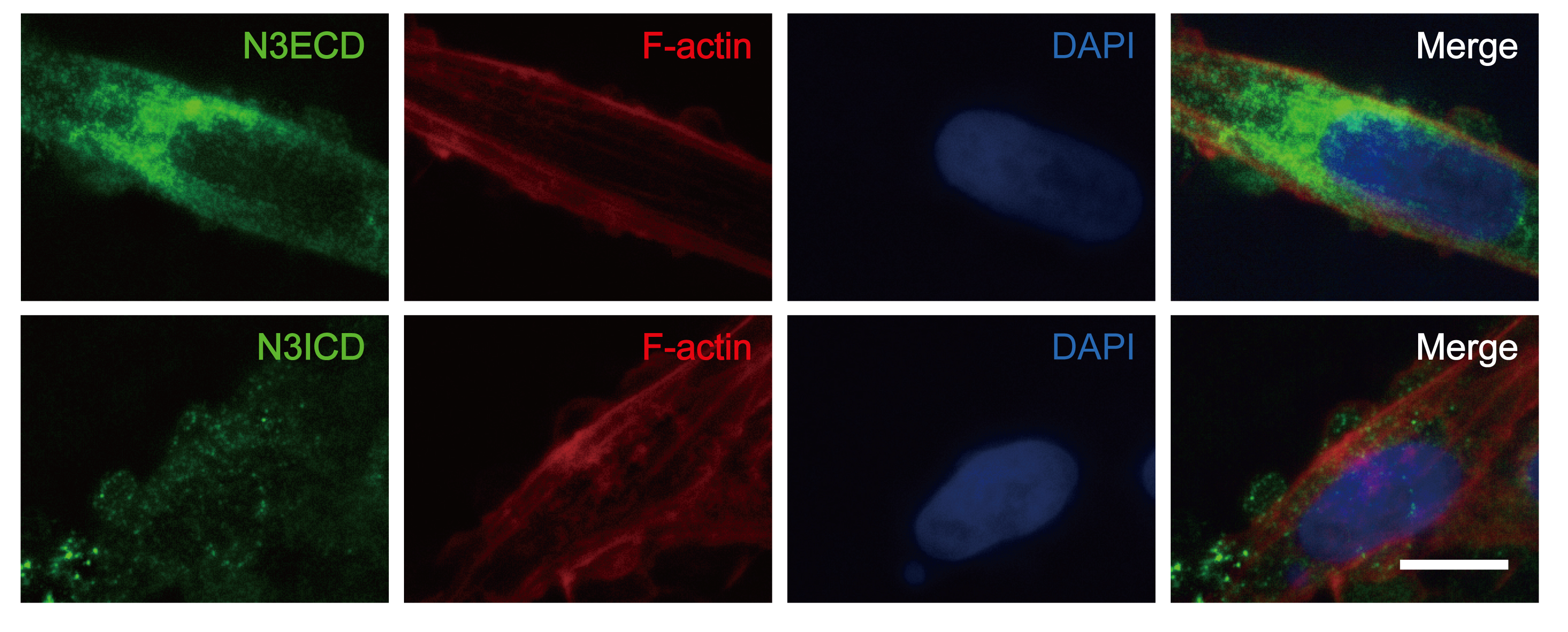

Supplement: Supplementary file 2 — Additional file 2: Figure S2. Representative images of N3ECD and N3ICD immunoreactivity and blebs. The bleb-like structure in CAD1–1 MC contained both N3ECD and N3ICD, but no more than the adjacent cytoplasm. Bar represents 5 μm. [file 13041_2020_573_MOESM2_ESM.jpg]

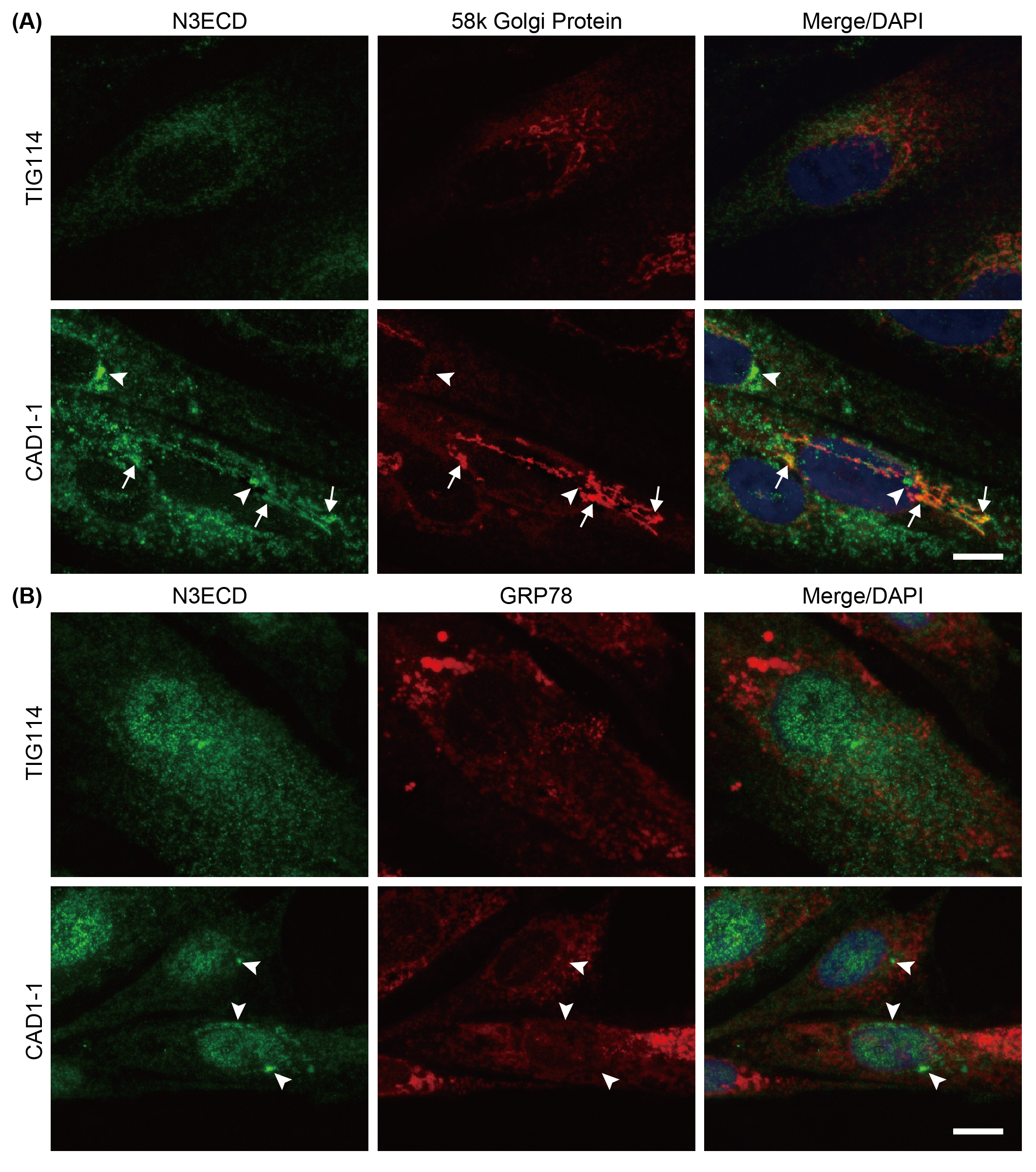

Supplement: Supplementary file 3 — Additional file 3: Figure S3. Representative images of N3ECD immunoreactivity. (A) While N3ECD were relatively uniformly distributed throughout the cell in controls (TIG114), more intense and aggregate-like immunoreactivity was unevenly distributed within the cell in CAD1–1, sometimes within the Golgi apparatus (arrows). Some of the intense staining were unassociated with Golgi apparatus marker (arrowheads) (B) Such strong focal immunoreactivity did not colocalize with an endoplasmic reticulum marker, GRP78 (arrowheads). Bars represent 10 μm. [file 13041_2020_573_MOESM3_ESM.jpg]

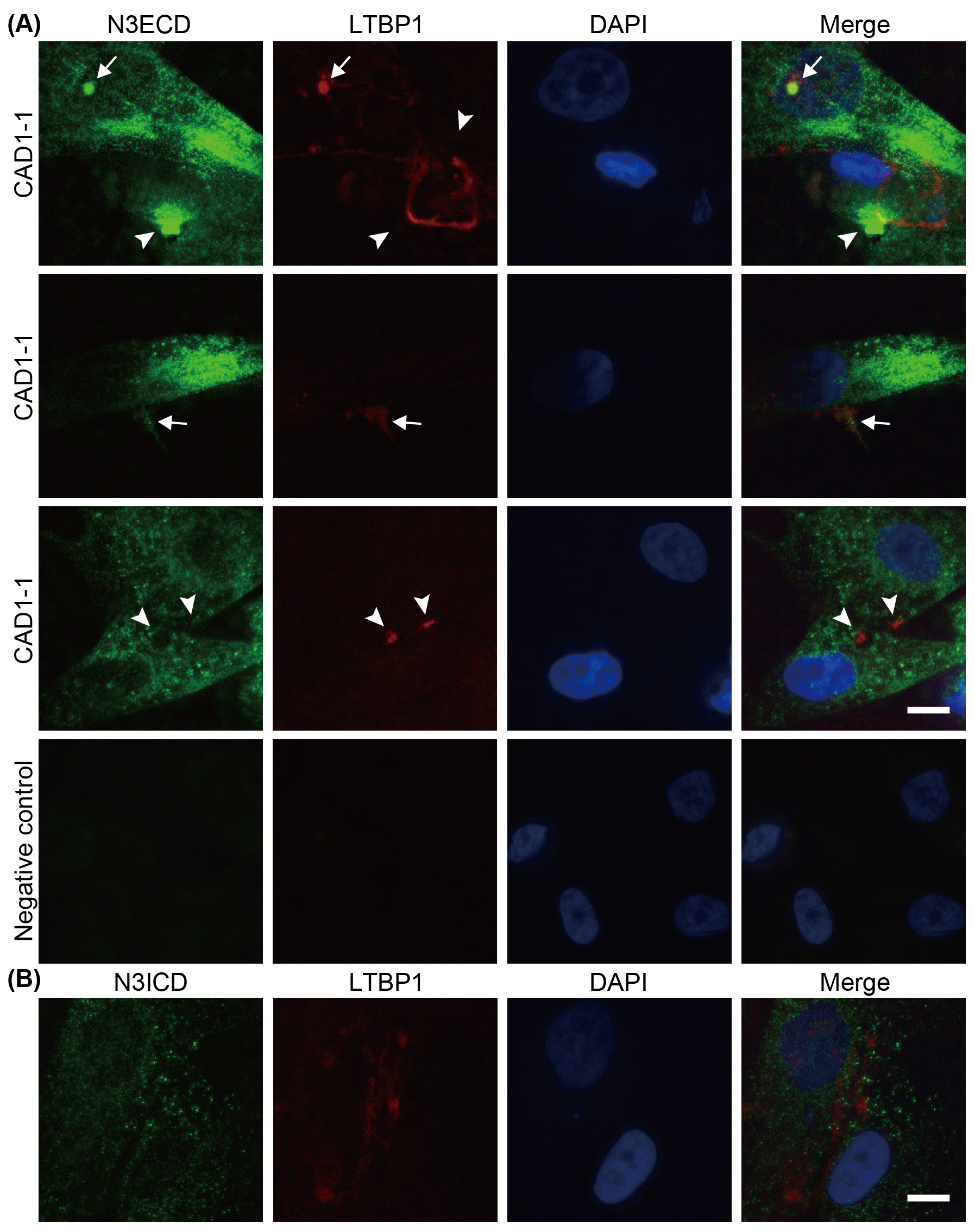

Supplement: Supplementary file 4 — Additional file 4: Figure S4. Colocalization of N3ECD and LTBP1 immunoreactivity. (A) Some of strong N3ECD immunoreactivity in CADASIL MCs were also positive for LTBP-1 (arrows) but the others were positive for either N3ECD or LTBP1 only (arrowheads). (B) LTBP1 did not colocalize with N3ICD immunoreactivity (B). Bars represent 10 μm. [file 13041_2020_573_MOESM4_ESM.jpg]

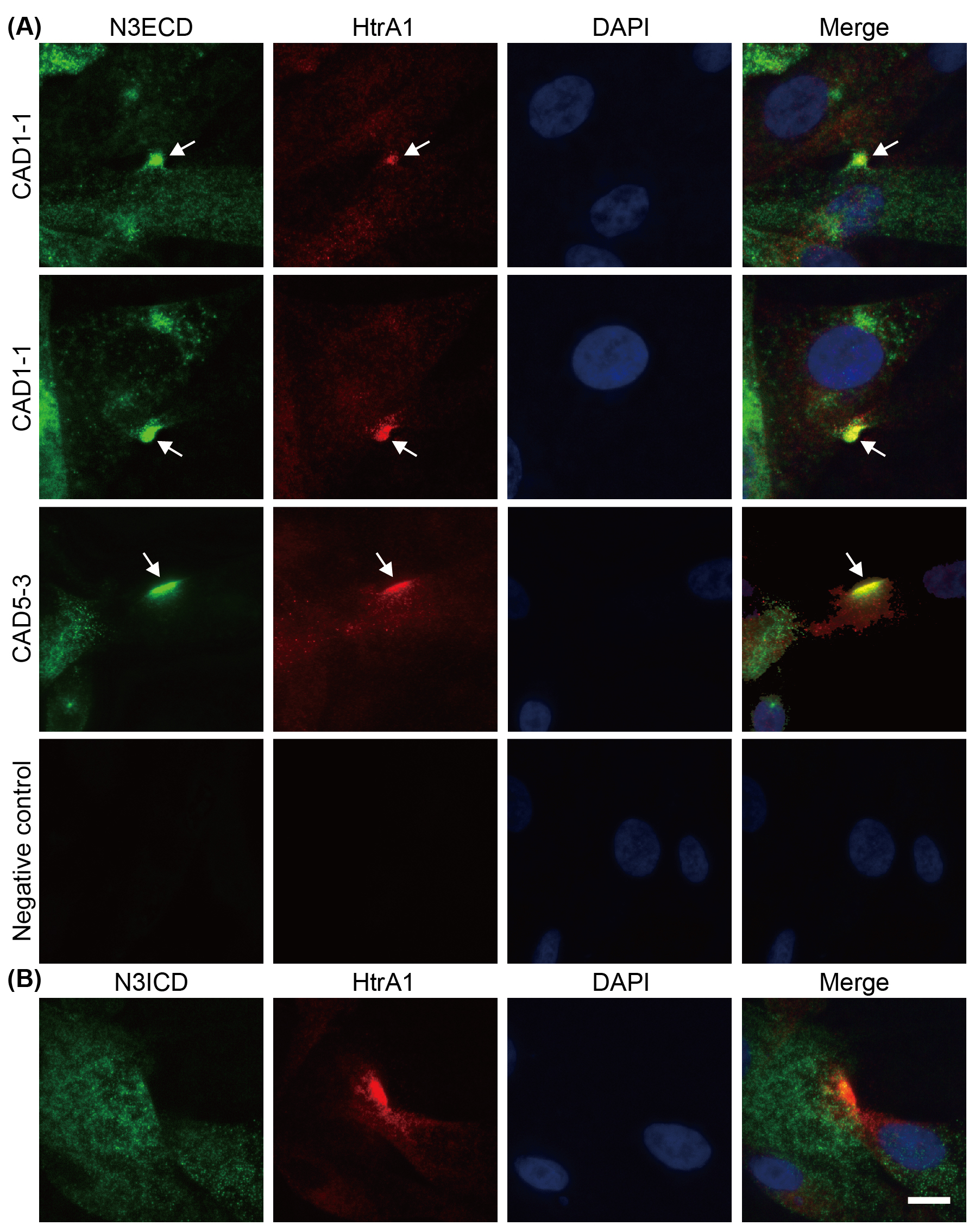

Supplement: Supplementary file 5 — Additional file 5: Figure S5. Colocalization of N3ECD and HtrA1 immunoreactivity. Intense HtrA1 immunoreactivity colocalized with N3ECD (A) but not with N3ICD (B). Bars represent 10 μm. [file 13041_2020_573_MOESM5_ESM.jpg]

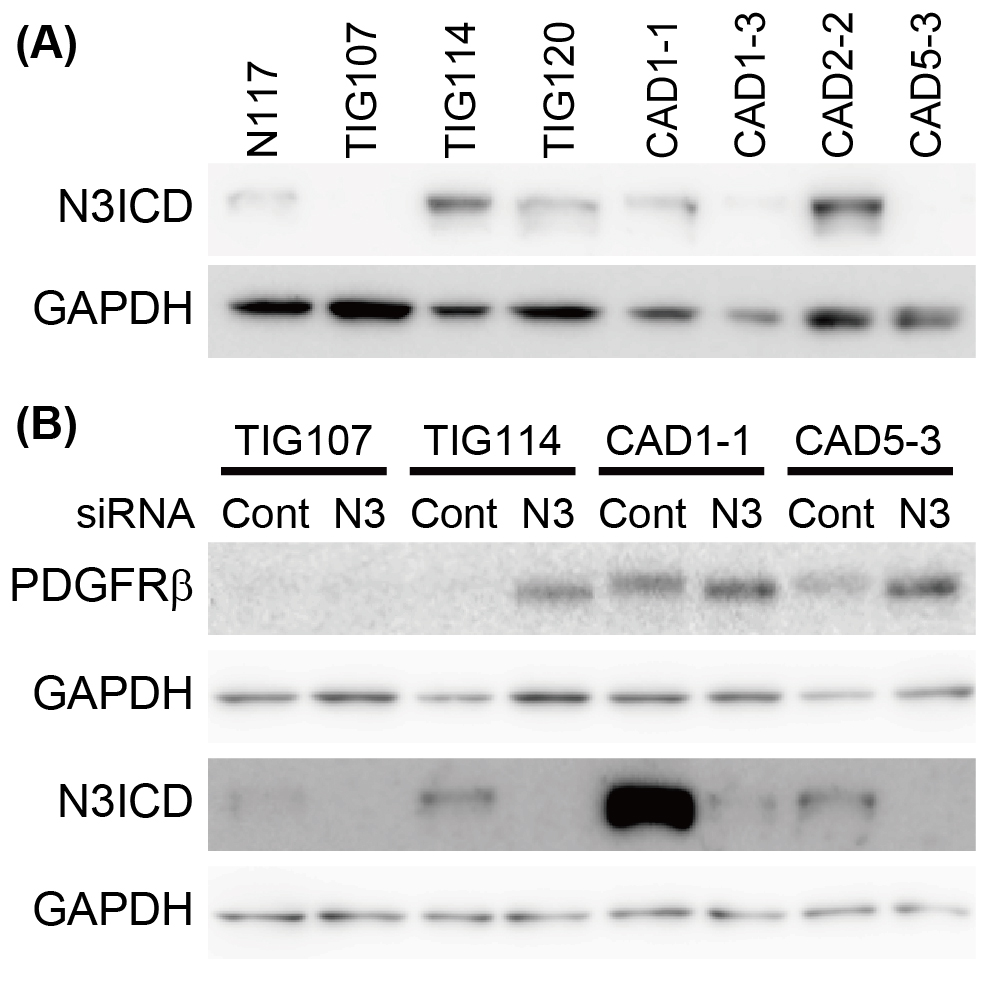

Supplement: Supplementary file 6 — Additional file 6: Figure S6. Expression of NOTCH3 and PDGFRβ in MCs. (A) The amount of N3ICD varied depending on the cell condition at the time of sampling and no consistent difference was found between control and CADASIL MCs. (B) Increased PDGFRβ was observed even after 7 days of NOTCH3 knockdown. [file 13041_2020_573_MOESM6_ESM.jpg]
